# EMI Shielding of the Hydrophobic, Flexible, Lightweight Carbonless Nano-Plate Composites

**DOI:** 10.3390/nano10102086

**Published:** 2020-10-21

**Authors:** Kanthasamy Raagulan, Jin Soo Ghim, Ramanaskanda Braveenth, Moon Jai Jung, Sang Bok Lee, Kyu Yun Chai, Bo Mi Kim, Joonsik Lee

**Affiliations:** 1 Division of Bio-Nanochemistry, College of Natural Sciences, Wonkwang University, Iksan City 570-749, Korea; raagulan@live.com (K.R.); pofuin@daum.net (J.S.G.); braveenth.czbt@gmail.com (R.B.); geuyoon@wonkwang.ac.kr (K.Y.C.); 2 Department of BIN Convergence Technology, Jeonbuk National University, Jeonju, Jeonbuk 54896, Korea; jjmoon@cands.kr; 3 Composite Research Division, Korea Institute of Materials Science, Changwon 51508, Korea; leesb@kims.re.kr; 4 Department of Chemical Engineering, Wonkwang University, Iksan 570-749, Korea

**Keywords:** nanoplate, EMI shielding, electrical conductivity, hydrophobic surface

## Abstract

The cost-effective spray coated composite was successfully synthesis and characterized by scanning electron microscopy, X-ray photoelectron spectroscopy, Raman spectroscopy, and X-ray diffraction techniques. The one step synthetic strategy was used for the synthesis of nanoplates that have a crystalline nature. The composites are amorphous and hydrophobic with micron thickness (<400 μm). The maximum contact angle showed by composite is 132.65° and have wetting energy of −49.32 mN m^−1^, spreading coefficient −122.12 mN m^−1^, and work of adhesion 23.48 mN m^−1^. The minimum thickness of synthesized nanoplate is 3 nm while the maximum sheet resistance, resistivity, and electrical conductivity of the composites are 11.890 ohm sq^−1^, 0.4399 Ω.cm^−1^, and 8.967 S.cm^−1^, respectively. The cobalt nanoplate coated non-woven carbon fabric (CoFC) possesses excellent sheet resistance, hydrophobic nature, and EMI shielding efficiency of 99.99964%. The composite can block above 99.9913% of incident radiation (X band). Hence, the composite can be utilized in application areas such as medical clothes, mobile phones, automobiles, aerospace, and military equipment.

## 1. Introduction

Electromagnetic interfering (EMI) shielding is a booming concern in the modern electronic environment. This is due to the rapid development of miniaturized, compact, highly integrated, and wireless electronic systems where hazardous electromagnetic radiation is being emitted. The emitted radiation not only affects human beings, but also causes the malfunction of adjacent electronic devices. The long-term exposure of radiation causes leukemia, cancer, mutation, headache, and nervous disorders. In addition, the EMI greatly influences the passage of migration birds and their habitants. When the human body is exposed to the EMI, this vibrates the body fluid and creates a dipole environment. The dipole environment leads to the dysfunction of organs, destruction of the function of the nervous systems, and destroys internal organs. Hence, minimizing the emission of electromagnetic radiation or achieving electromagnetic compatibility (EMRC) is a crucial need. EMRC is the concept where the function of the electronic devices is unchanged in the EMI environment. The EMI shielding effect (EMI SE) is expressed in dB and other parameters such as specific EMI SE and absolute EMI SE, depending on the thickness and density of the shielding materials, respectively [1,2,3,4,5].

To achieve EMRC, scientists around the globe use various materials such MXene, graphene, graphite, carbon nanotube, metal nanomaterials, and polymer. Conventionally, metal plates have been used as shielding materials. However, modern electronics need to have a light weight, flexible, thinner composition, for which nanomaterials are mixed with a proper binder. Generally, polymers are used as binder, and exceptionally conductive polymers are being used as EMI SE components as well. Further, the EMI shielding ability of the composite can be tuned by changing the constitutional elements of the composite and the structure of the composite which depends on the fabrication process used. There are many fabrication processes such as spray coating, dipped coating, filtration process, spin coating, freeze drying, and solvent casting. Nevertheless, post treatment of the composite significantly affects the EMI shielding of the composite [6]. 

The basic mechanism of EMI shielding consists of absorption, reflection, multiple reflection, and transmission. The reflection depends on the electrical conductivity of the shielding materials while absorption depends on the structural features, dipole, and thickness of the composite. However, many other parameters also influence EMI SE, and those are wave impendence of the air and shielding materials, relative magnetic and electric permeability, propagation constant of the wave, skin depth, relative magnetic, and electric conductivity, refractive index, angular frequency of the wave, and transmission coefficient. On the whole, most of the shielding behavior of the composite by absorption with multiple reflections can be neglected. MXene shows excellent EMI shielding behavior compared to other shielding materials which is due to the highest electrical conductivity and the surface functional groups. A recent study showed that the intercalation of the silver nanowire with MXene and other nanomaterials significantly improve the EMI shielding of the composites [3,4,5,6,7,8,9].

In this study, we developed a cheapest strategy to synthesize metal nanoplate and fabrication of the corresponding composites. Nonwoven carbon fabric (NWCF) was coated with corresponding nanoplates and it was characterized by using XPS, XRD, and SEM. In addition, its hydrophobic nature and EMI shielding are also studied in detail. The composite denoted as CuFC, CoFC, FeFC, ZnFC, and NiFC in which metals denoted as nanoplates and FC implied the nonwoven carbon fabric. The Co based composite showed excellent EMI SE and the composite blocked 99.99% of incoming electromagnetic radiation (X band).

## 2. Materials and Methods

### 2.1. Materials

Cetyl trimethylammonium bromide (CTAB) (98%), *N*,*N*-Dimethylformamide (DMF) (99.5%), Polyvinylpyrrolidone (PVP) (86–89%), polyacrylamide (PAM), Sodium borohydride (NaBH_4_) (96%), anhydrous (Copper chloride (CuCl_2_) (97%), cobalt chloride (CoCl_2_) (97%), nickel chloride (NiCl_2_) (98%), zinc chloride (ZnCl_2_) (98%), and ferrous chloride (FeCl_2_) (98%) were purchased from Sigma Alrich (Seoul, Korea). The Poly(vinylidene fluoride) (PVDF) was obtained from Alfa Aesar (Seoul, Korea) and sodium dodecyl sulfate (SDS) (95%) was issued by Samchun chemical (Seoul, Korea). Polyethylene terephthalate (PET) binder (fiber diameter 2.2 dtex, 5 mm), and carbon fiber (fiber diameter 7-micron, 6 mm length) were collected from TORAY product, (Tokyo, Japan). All the chemicals were utilized without further purification.

### 2.2. Synthesis of the Cu Nanoplate

An equal amount of SDS and CTAB (1 g L^−1^ each) were stirred in deionized (DI) water for 2 h. Then, CuCl_2_ (0.05 M) was added to the above solution and stirred for 1 h. The cold 0.2 M of NaBH_4_ solution was drop wisely added while stirring. The resultant mixture was further stirred for 24 h and washed well with deionized (DI) water at filter. The product was dried in a vacuum oven at 50 °C overnight. The same procedure was repeated to synthesis Co, Zn, Fe, and Ni nanoplates. 

### 2.3. Nonwoven Carbon Fabric Synthesis by Wet-Laid Method

The non-woven carbon fabric was synthesized according to the method reported by Raagulan et al. [M1]. Briefly, 600 g of carbon fiber, 150 g of PET binder, and 0.3 weight percent of dispersant (PAM) were dispersed in a sufficient amount of deionized (DI) water at 500 rpm for 10 min. The web was produced by a general wet laid method during which the drum dryer surface was kept at 140 °C with the heating rate of 7 m min^−1^. The areal density of obtained fabric is 30 g m^−2^ [4]. 

### 2.4. Composite Preparation

The nanoplate (Cu, Co, Fe, Zn, Ni) and PVDF were taken with a ratio of 2:1 and mixed in DMF (3 g L^−1^). The resultant mixture was stirred for 24 h and spray-coated on 15 × 15 cm^2^ of nonwoven carbon fabric (NWCF). The drying process was done by using air gun. The composites were coated 15 times and denoted as CuFC, CoFC, FeFC, ZnFC, and NiFC. 

### 2.5. Characterization

A field emission scanning electron microscope (SEM, S−4800 (Hitachi, Tokyo, Japan) was used to examine the surface morphology and cross-section of the composites. XPS with a 30–400 µm spot size at 100 W of Emax (Al anode) (K-Alpha, Thermo Fisher, East Grinstead, UK) was used to analyze the chemical environment and elemental percentage of the composites. A high-power X-ray diffractometer D/max-2500V/PC, (Ragaku, Tokyo, Japan) with Cu(K) was utilized to record the X-ray diffraction patterns of the nanoplates and composites. The structural features of the nanoplates and composites were explored using a high-resolution Raman spectrophotometer (Jobin Yvon, LabRam HR Evolution (Horiba, Tokyo, Japan). A Mitutoyo thickness 2046 S dial gage (Mitutoyo, Kanagawa, Japan) was used to measure the thickness of the composites. The X-band (8.2–12.4 GHz) EMI shielding was measured using a vector network analyzer (VNA, Agilent N5230A, Agilent Technologies, Santa Clara, CA, USA) with a sample size of 22.16 × 10.16 mm. The wetting ability of the surfaces was measured by using a contact angle meter, Phonix-300A (S.E.O. Co., Ltd., Suwon, Korea). The four-probe method FPP-RS8, DASOL ENG (Seoul, Korea) was used to obtain the electrical conductivity of the composites.

## 3. Results and Discussion

### 3.1. Scanning Electron Microscopic Analysis (SEM)

**Figure 1:**
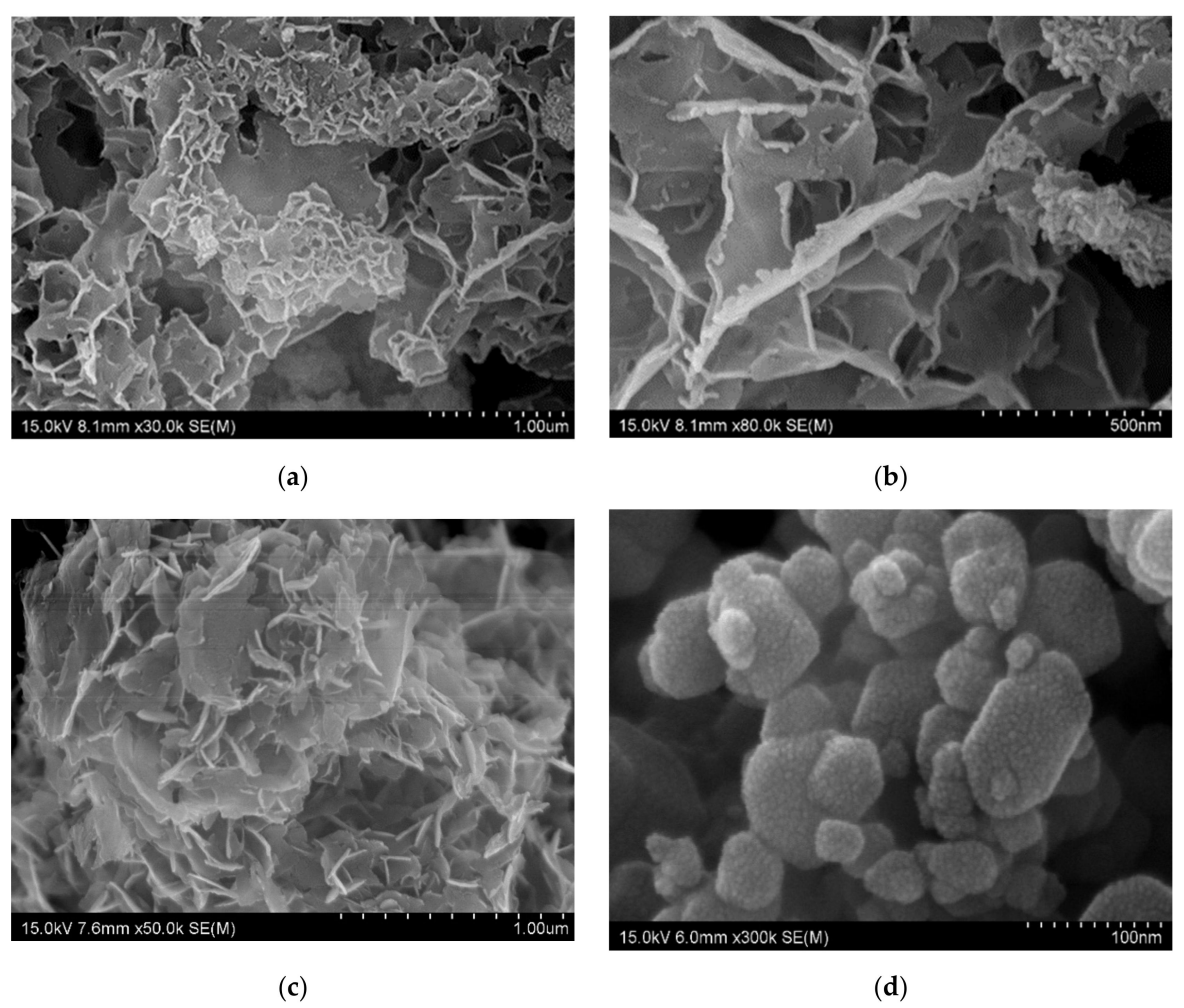

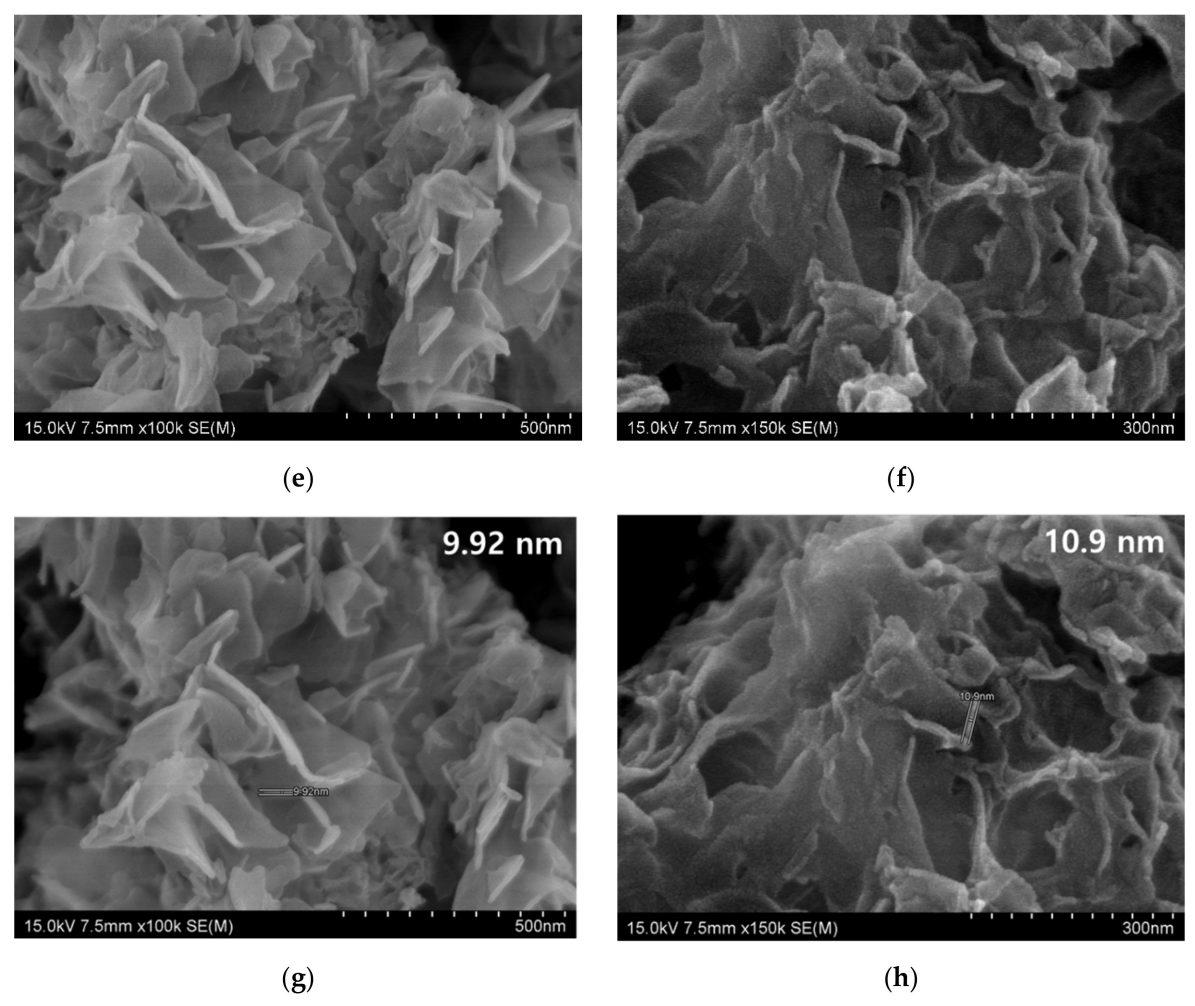
SEM image of nanoplate (**a**) Co (×30,000), (**b**) Co (×80,000), (**c**) Fe (×50,000), (**d**) Cu (×300,000), (**e**) Zn (×100,000), (**f**) Ni (×150,000), (**g**) thickness of Zn (×100,000), and (**h**) thickness of Ni (×150,000).

**Figure 2:**
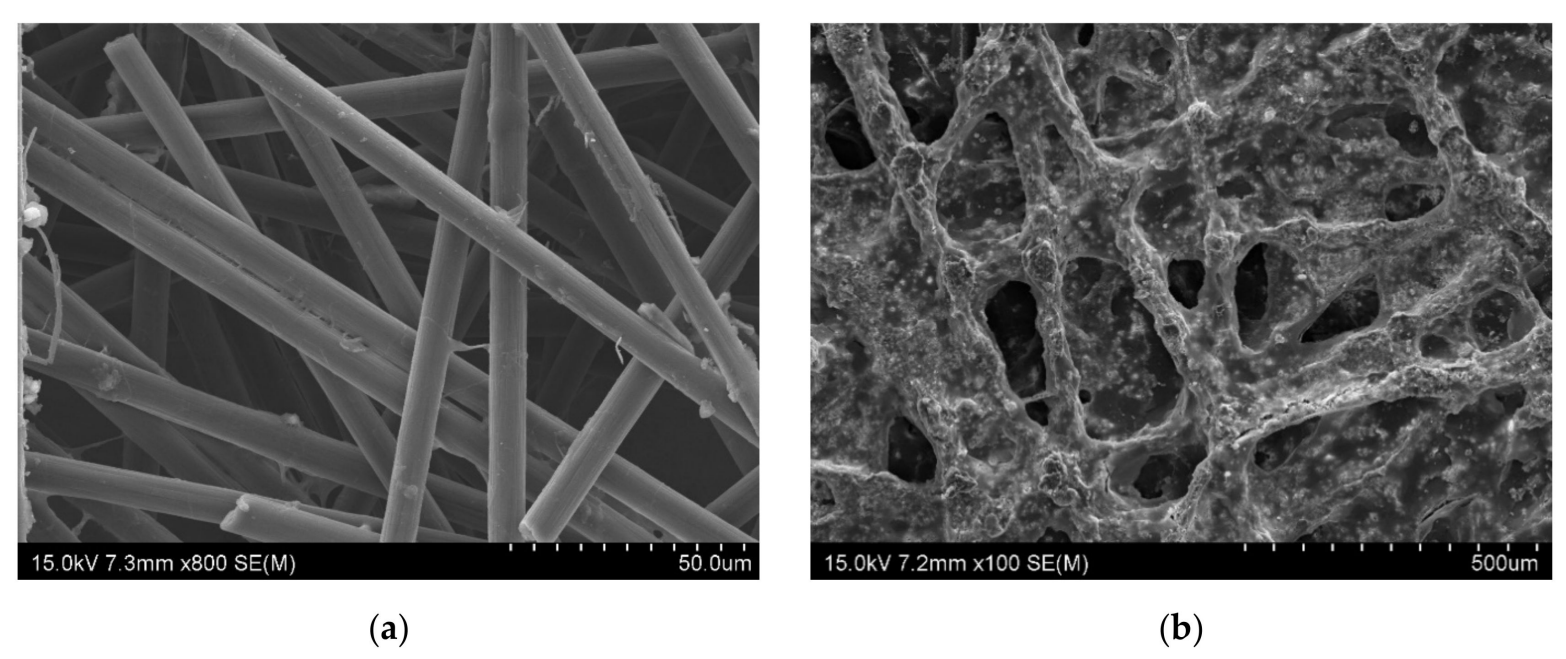

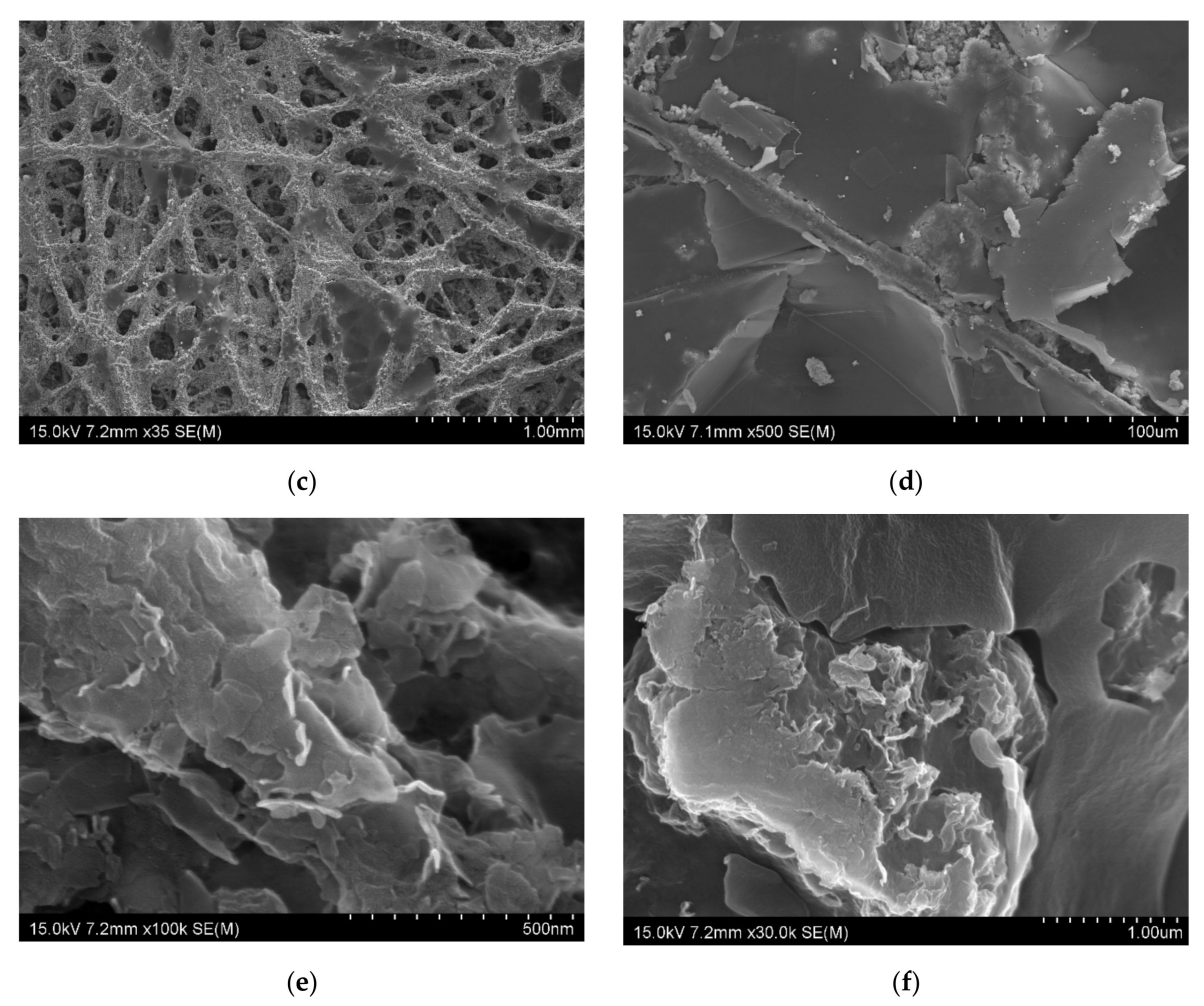
SEM image of the composites (**a**) CF (×800), (**b**) CoFC (×100), (**c**) FeFC (×35), (**d**) CuFC (×500), (**e**) ZnFC (×100,000), and (**f**) NiFC (×30,000).

The SEM is the tool used to analysis the morphology of materials, such as nanomaterials, composites, fabrics, and two-dimensional materials, as well as the topography of the materials [5]. The morphology of the metal nanoplate showed in Figure 1, and each of them has distinguished structural features (Figure 1a–f). The Co, Fe, Zn, and Ni nanoplates commonly possess coral like structure, and each differ slightly (Figure 1a,c,e,f). The Co nanoplate showed curling edge and the aggregation of Co nanoplates implied the porous structure (Figure 1b). In addition, Co nanoplate exhibits similarity of exfoliated MXene [5]. However, Fe, Zn, and Ni don’t have prominent curling edges while Cu was shown to have a hexagonal shape. It is obvious that the Cu hexagonal nanoplate is made of many small Cu nanoparticles (Figure 1d). Moreover, Fe and Zn nanoplate have similar structural feature and appearance seems like fish scale [10]. The fish scale nanoplates aggregate and form a coral like structure. The Ni nanoplate morphology slightly varies from others which is due to the thickness and size of Ni nanoplate, which leads to a porous nature. On the whole, it is apparent that all the nanoplates were synthesized in the same condition and holding different morphology. This indicates that each metal ions act as self-catalyst and form with various morphological feature without changing the dimension (2D). The morphology of the pure metal nanoplate changed after the coating on the surface of the carbon fabric which is because of PVDF and carbon fiber in the fabric (Figure 2). It is apparent that the thickness of the synthesized nanoplates lie in the range of 3–18 nm which are in the nanoscale and the thickness of the nanoplates such as Zn, Ni, Fe, Co, and Cu are 9.92, 10.9, 8.87–18, 3, and 7–10 nm, respectively. In addition, the size of the one nanoplate is about 500 nm which is the advantage for the EMI shielding (Figure 1a–h, and Section 3.2). According to the Bian et al. the thickness of single layer MXene is 4 nm which is similar to that of synthesized Co nanoplate. This gives rise the notion that the MXene without surface functional groups replaces the Co nanoplate [11]. The thickness of the single layer graphene is 0.335 nm while 3.185 nm thickness showed by 7-layer graphene [12]. Hence, the metal nanoplate can be replaced by multi-layer graphene and MXene. The MXene, and graphene synthesis are multistep process need more time, cost, various instruments, and chemicals, However, metal nanoplate synthesis is single step process [11,12]. In addition, nanoplates-polymer composites are better EMI shielding material than MXene and graphene polymer composition. Furthermore, metal nanoplates shows excellent EMI shielding than nanoparticle (Fe, Ni, Fe_3_O_4_). Thus, nanoplates can substitute the nanoparticle-based EMI shielding materials and enhance EMI shielding properties of the composites. Conventionally, metal used as EMI shielding material and nanoplate eliminate the drawback of metal plate, such as weight and thickness of the equipment. As we have reported already about graphene-based fabric composite exhibit less shielding ability than that of metal nanoplate based composites (Section 3.6.2) [13].

The morphology of the composites shown in the Figure 2a–f. In the nonwoven carbon fabric, the fibers arranged irregular manner with some parallel alignment. The gabs and grooves are common in the non-woven carbon fabric web. Further, the fibers in non-woven carbon fabric have cracks and defect which is the inherent nature, and these cracks and defects can be minimized by introducing the foreign nanomaterials such as graphene, MXene, carbon nanotube, nanoparticle, metals and polymers [4]. In this study, we used nanoplates which is similar to graphene and MXene to coat NWCF, and we compare EMI shielding effects of different nanoplate composites. The CoFC has pores, covered by Co nanoplate coated carbon fibers and the Co-PVDF composition increases the surface roughness of the composite (Figure 2b). Moreover, the FeFC has similar structure as CoFC. It is obvious that the nanoplate-PVDF composition form plate like structure which is the main reason for the higher EMI shielding effect of the composites and have cracks that is the drawback for the better EMI shielding. In addition, the higher size nanoplate facilitate electron mobility in the composite which increase the electrical conductivity, and interconnection of nanoplate, therefore, enhance the EMI shielding (Figure 2d). This is because the cracks constrain the electron mobility in the composite and reduce the EMI shielding of the materials (Figure 2d–f) (4–5, 10). The nanoplates cover the fiber layer by layer which increase the electric conductivity, EMI SE, and other parameters. The coating of the nanoplate changes the properties of the NWCF which is obvious in the XRD analysis (Section 3.3). The structural feature and its effect on EMI shielding is discussed in the Section 3.6.

### 3.2. X-ray Photoelectron Spectroscopy (XPS) Analysis

**Figure 3:**
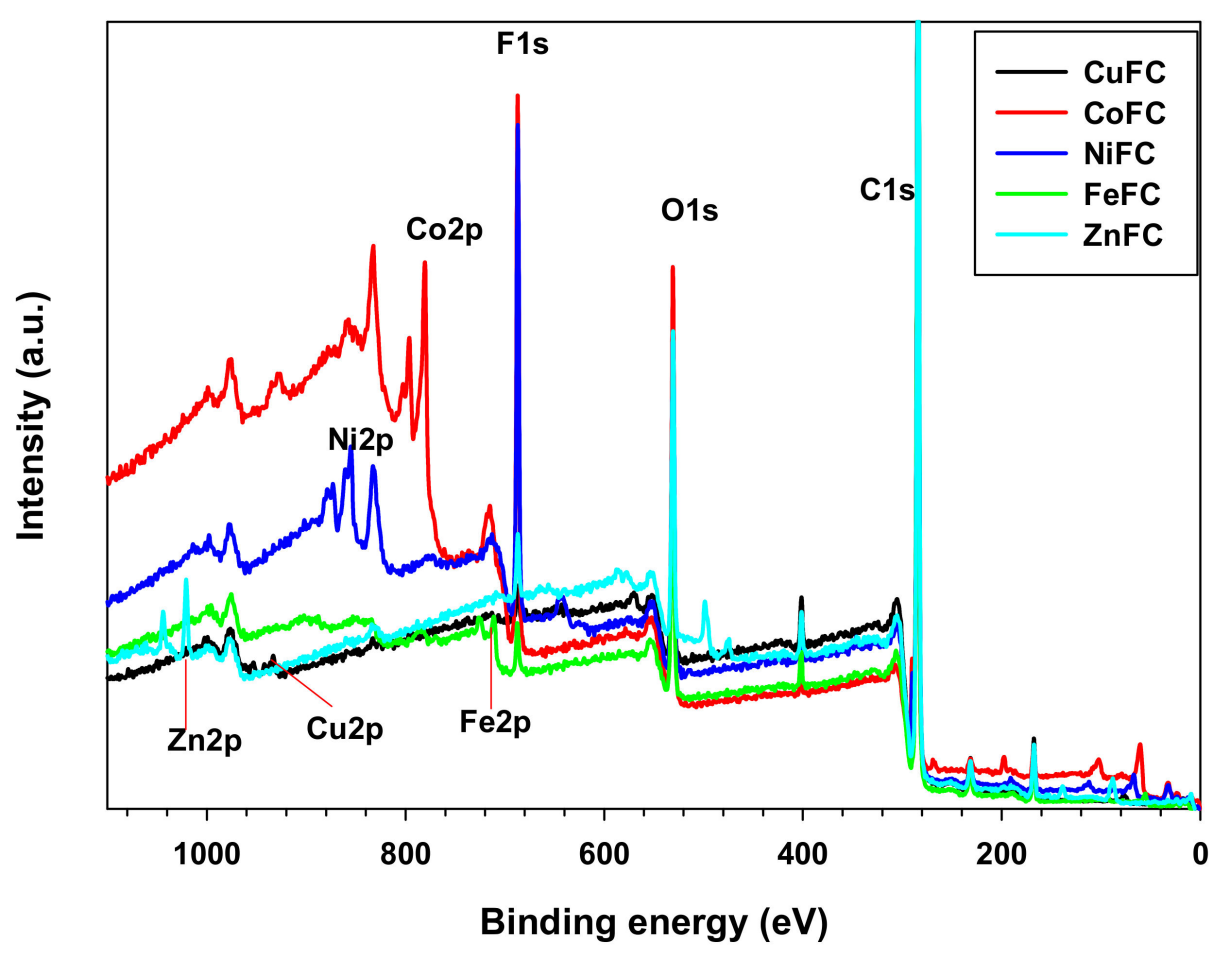
XPS survey of the composites.

XPS is the useful tool to investigate structural features, elemental composition, and surface functional groups of the composites, because different elements, functional groups, and chemical centers have a specific binding energy and chemical shift through which the corresponding composition can be confirmed. Figure 3 shows different elements and its corresponding binding energy. In addition, the Table 1 exhibits the elemental percentage of the composition. The carbon is the major component as nano-plates were coated on nonwoven carbon fabric while oxygen is the second major element present in the composite which originates mainly from carbon fabric and metal oxides formed during the composite processing. The surfactants have almost similar percentage and confirmed equal amount of the SDS and CTAB were used. The PVDF is the main Florine source and metals came from corresponding metal nano-plates. Most of the elements such as C1s, O1s, S2p, F1s, and N1s are at their specific binding energy position in all the composite and corresponding binding energies are about 284, 530, 167, 687, and 401 eV, respectively. In addition, the binding energy of the metals such as Zn, Fe, Ni, Co, and Cu are located at 1020.83, 711.18, 855.55, 780.77, and 933.23 eV, respectively. It is obvious that the introduction of the metal nanoplates do not affect the binding energy position of the other elements in the composite [13]. The 933.23 eV for Cu indicates that the Cu nanoplate consist Cu and copper (II) oxide [14]. According to the Al-Kuhaili et al. the NiFC composite consist Ni-O functionalities which indicates that the air gun drying caused slight oxidation of Ni nanoplate [15]. A binding energy of Fe2p located at 711.18 eV specifies that the form iron oxide has Fe^3+^ while the Zn2p position implies the presence of Zn-O, and Zn-N functional groups which are from oxidation and CTAB [16,17]. Biesinger et al. reported that the binding energy position of Co in the composite indicates that it consists Co-O functional groups which is due to oxidation of Co nanoplate (Figure 3 and Table 1) [18]. The used nanoplate slightly oxidized and remaining came from nonwoven carbon fabric. Thus, all the composites have C, O, F, S, and N and corresponding metal. The presence of S and N indicate that the SDS and CTAB attached with the nanoplate, which created a dipolar environment. Therefore, these metal nanoplates can be dispersed both polar and nonpolar materials. This phenomenon gives rise the homogenized mixture both polar and nonpolar environment. This is the advantage of the nanoplates and enhance the EMI shielding as well.

### 3.3. X-ray Diffraction Spectroscopy (XRD) Analysis

**Figure 4:**
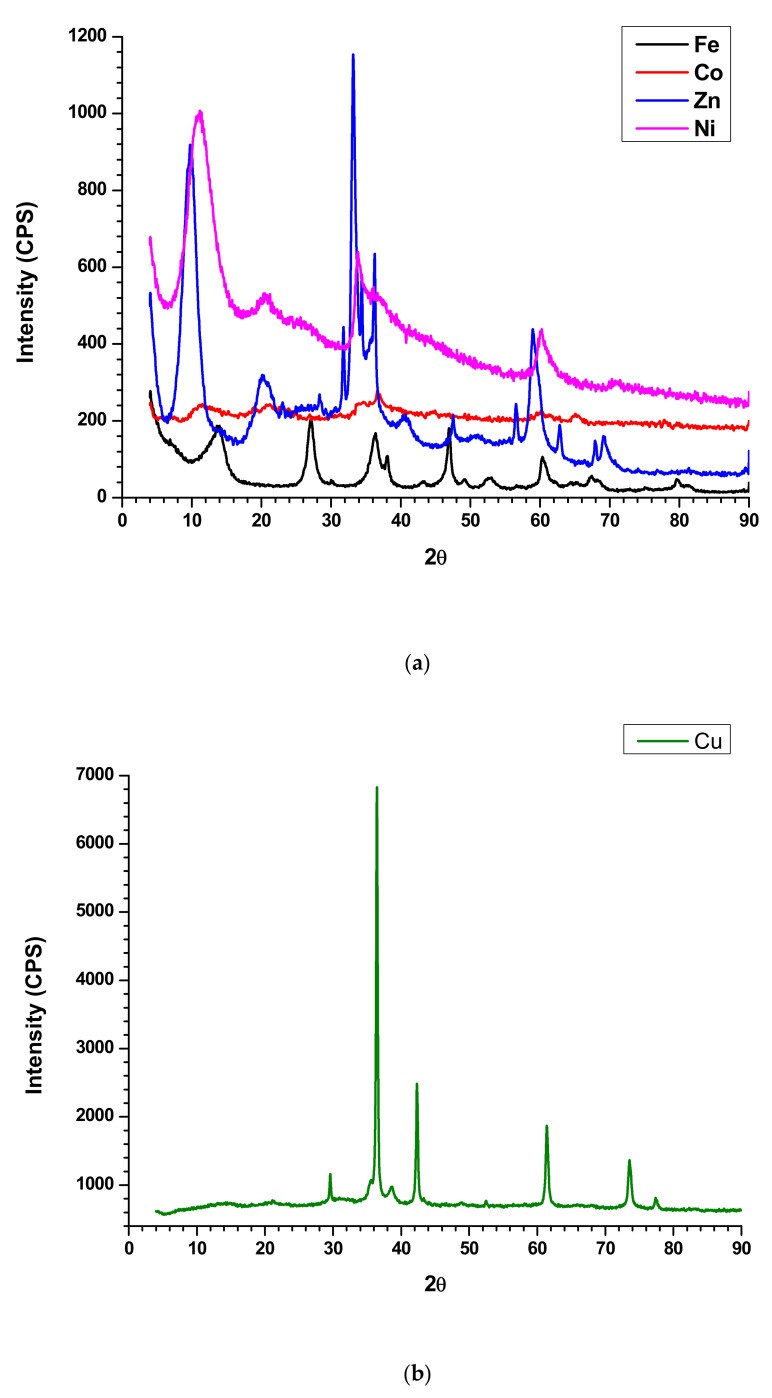

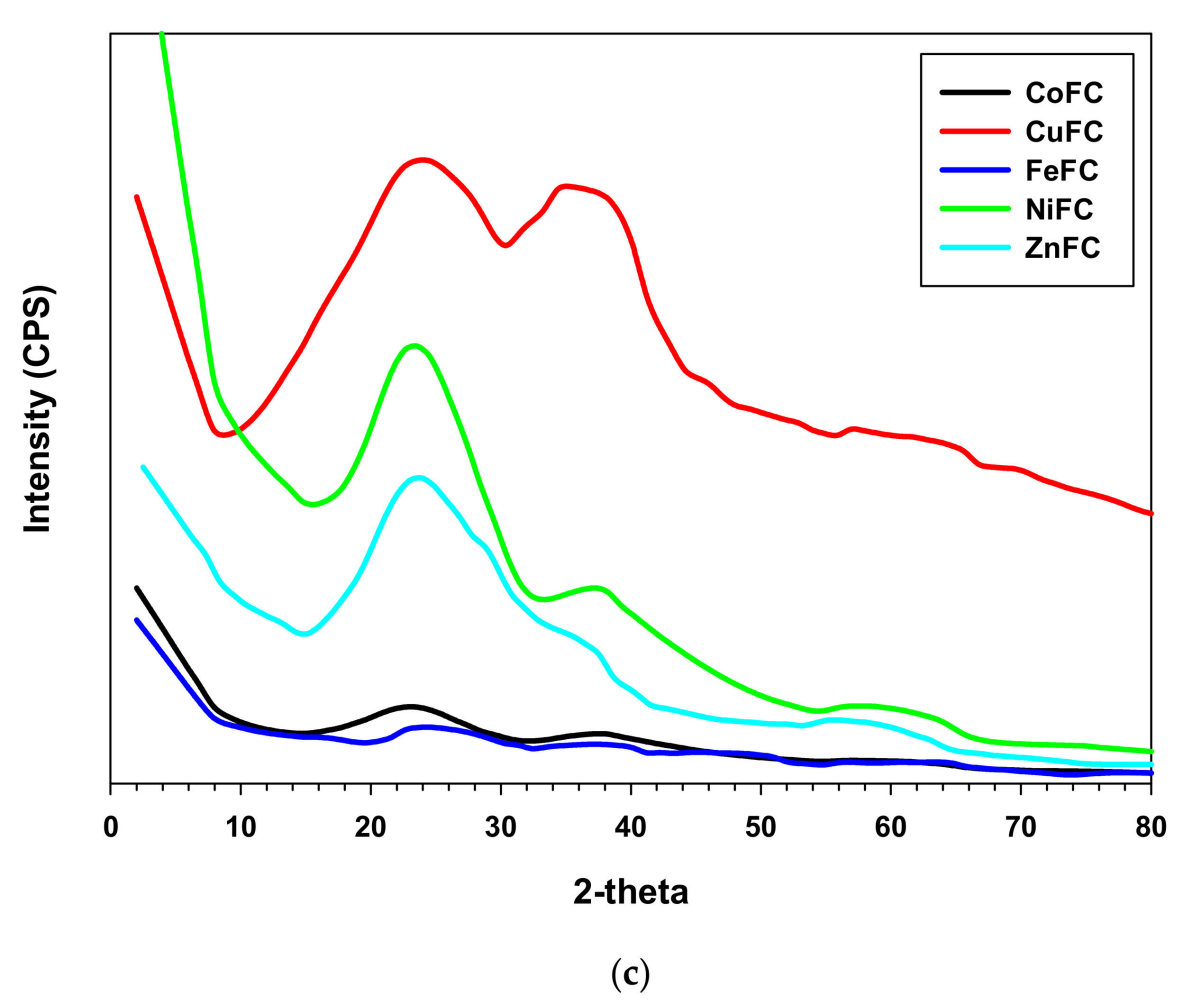
XRD of (**a**,**b**) nanoplates, and (**c**) composites.

XRD profile is utilized to evaluate the crystalline or amorphous nature of the composite. The sharp peaks indicate the crystalline nature of the compound while broad peaks specify amorphous materials. It is obvious that the synthesized nanoplates are crystalline in nature and exhibits sharp peaks in XRD profile. The Ni nanoplates show 2θ peaks at 11.33°, 20.62°, 33.76°, 36, 60.23°, and 70.81° while Zn nanoplates exhibit Bragg’s angle at 9.71°, 20.29°, 23.05°, 28.33°, 31.86°, 33.14°, 34.43°, 36.33°, 40.66°, 47.57°, 56.52°, 58.95°,62.95°, 67.9°, and 69.19°. In addition, Co nanoplate displays 2θ peaks at 11.48°, 19°, 21.29°, 27.1°, 31.38°, 34.71°, 36.67°, 45°, 60.23°, 65.19°, and 77.71° whereas the 2θ peaks at 6.86°, 13.71°, 27.19°, 30.1°, 36.33°, 38.1°, 43.38°, 46.90°, 49°, 53°, 56.71°, 60.38°, 64.57°, 65.33°, 67.43°, 75.29°, 79.76°, and 81.23° represent Fe nanoplate. The Cu nanoplate exhibits sharp 2θ peaks at 29.65°, 36.47°, 38.69°, 42.35°, 48.78°, 52.6°, 61.4°, 73.6°, and 77.37° (Figure 4a,b). The 2θ peaks exhibited by nanoplates are the characteristic peaks by which each nanoplate can be identified. According to the XRD profile of the composites displayed amorphous in nature. In addition, the composites are a mixture of amorphous and crystalline components and their ratio is different for each other. The Bragg’s angle at 23.52°, 36.56°, and 61.45° are same for the all the composite while ZnFC exhibited additional peak at 58.39°. Further, all the composite such as CuFC, NiFC, ZnFC, FeFC, and CoFC showed a different lower characteristic peak at 8.78°, 15.72°, 15.05°, 12.84° and 12.84°, respectively, which is the characteristic peak for the composites (Figure 4c). The 2θ peak at 23.52° is due to the NWCF which implies the presence of graphite structure. In addition, PVDF gives rise 2θ peaks at 17.7° and 20.4° which is not prominent indicates that the less amount of PVDF was used for the composite preparation [13]. In addition, the 2θ peaks at 36.56°, and 61.45° are the characteristic peaks of the all the composite fabricated in this study. CuFC exhibits 2θ peaks at 36.4°, 52.5°, 64.5°, and 76.8° imply presence of corresponding reflective planes of Cu nanomaterials such as (110), (200), (113), and (220), respectively [19]. The 2θ peaks of CoFC at 38.3°, and 62. 3° are generated by reflective planes of (222), and (440), respectively which approves existence of Co [20]. The FeFC composite reveals the amorphous nature with various 2θ peaks at 37.8°, 48.5°, 64.3°, and 82.1° assure the Fe and Fe_2_O_3_ which agreed with XPS analysis [21,22]. The reflective plane (220) at 58.8° accepts the occurrence of the Ni in the NiFC while ZnFC exhibits characteristic feature peak at 55.3° [23,24]. In addition, the disappearance of characteristic peaks of nanoplate in the composites confirm the effective bonding between nanoplate and PVDF. Further, the broad peaks of the composites in the XRD profile indicate that the crystalline nanoplate composition becomes amorphous in nature. 

### 3.4. Raman Spectroscopy Analysis

**Figure 5:**
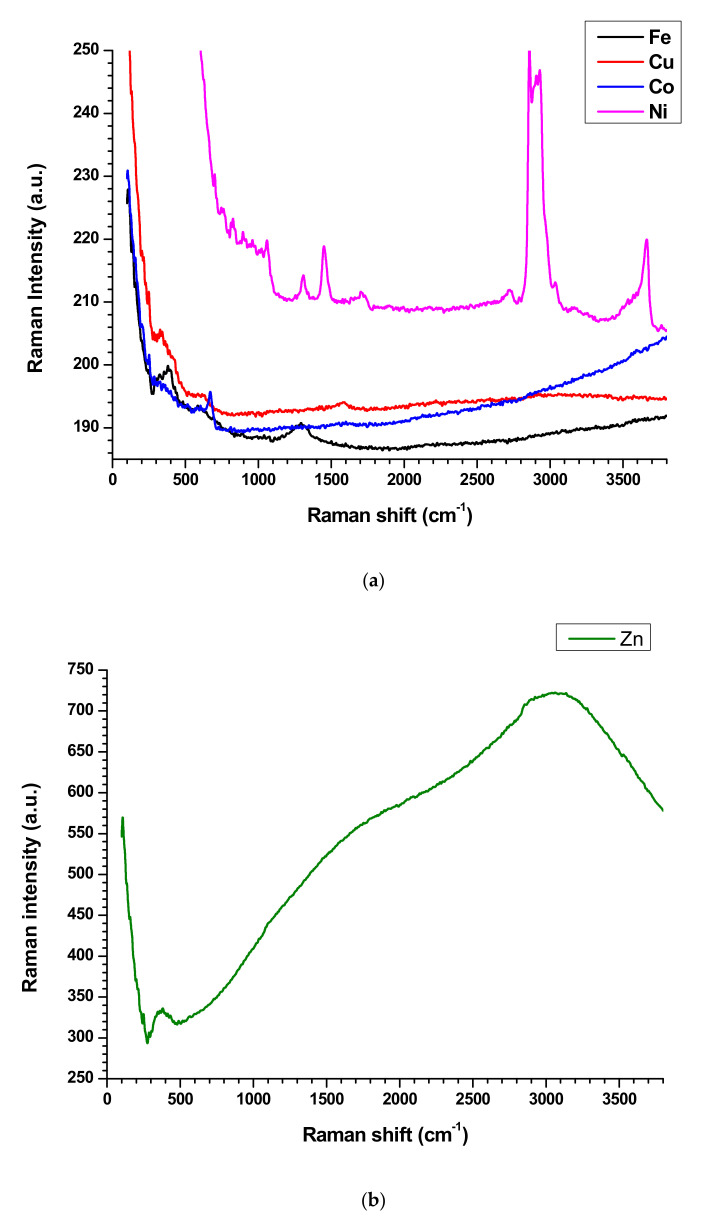

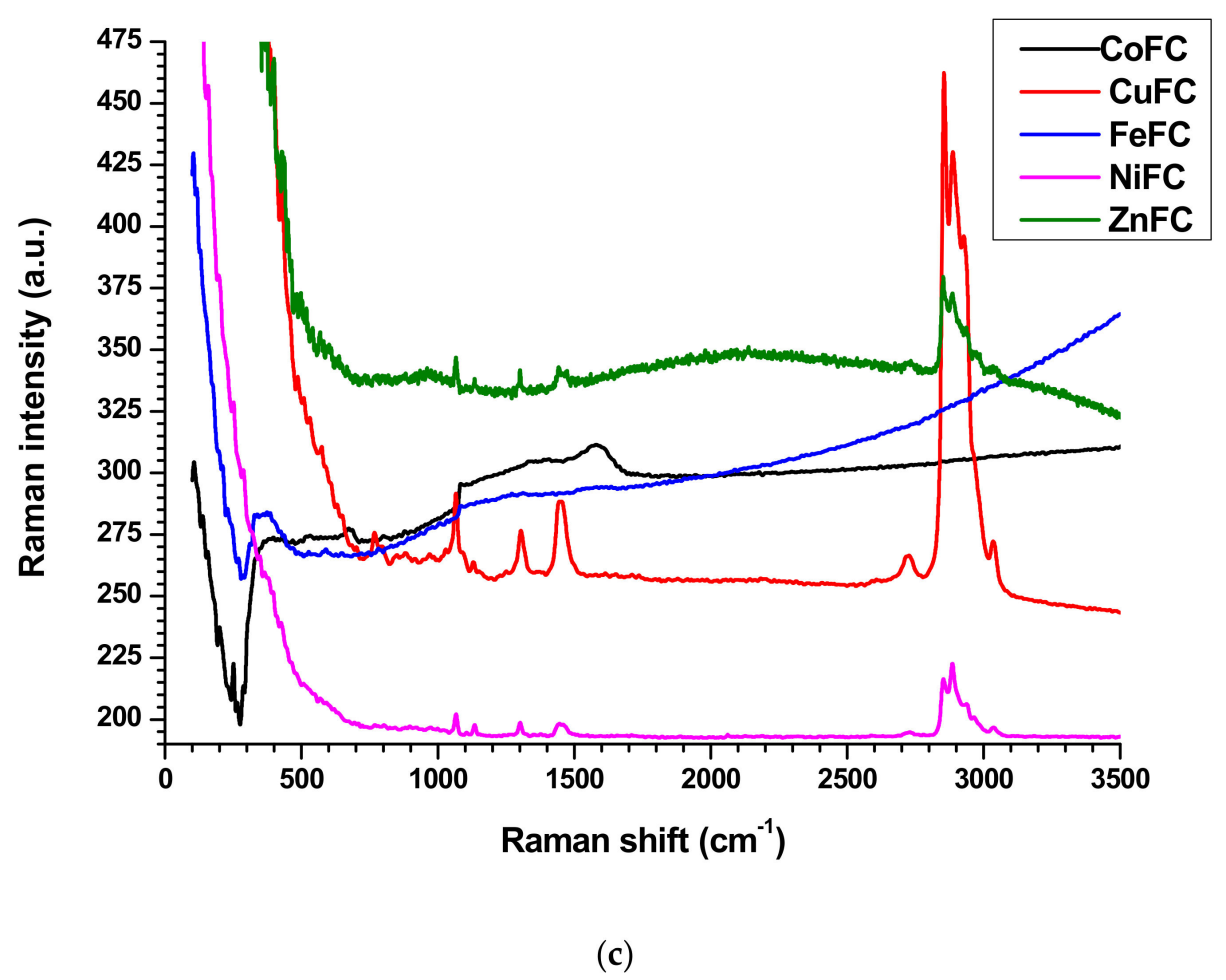
Raman spectroscopy of (**a**,**b**) nanoplates, and (**c**) composites.

The Raman spectroscopy analysis is a non-destructive technique, is used to investigate physiochemical behavior, crystalline nature, level of defects, and structural feature of the materials [4,5,6,13]. The nanoplates have downward and upward peaks which are specific for each nanoplate. The Zn, Fe, and Co nanoplates exhibit downward peaks at Raman shift of 276.36, 276.36, and 549.78 cm^−1^, respectively. Further, Cu nanoplates exhibit downward Raman shift at 299.39, 985.36, and 1029.78 cm^−1^ while Ni nanoplate shows non. The Zn nanoplate shows Raman shift at 371.77 and 3046.58 cm^−1^ while Raman shift at 381.64, 582.33, and 1296.28 cm^−1^ represent Fe nanoplate. The Raman shifts at 327.36, 526.41, 1589.1, 2220.78, and 2913.33 cm^−1^ denote the Cu nanoplate whereas designated peaks at 251.1, 673.16, 818.18, 1599.56, and 3603.89 cm^−1^ designate the Co nanoplate. However, Ni nanoplates behave differently that is in the range of 704–1058 cm^−1^ displays many small peaks (704, 758.35, 830.73, 891.6, 963.98, 1019.91, 1057.75) cm^−1^ which are characteristic peaks of Ni nanoplates) and following Raman shift further confirms the Ni nanoplates such as 1307.79, 1445.97, 1705.88, 2724.15, 2857.7, 2908.39, 2929.78, 3035.1, 3173.24, 3666.75, and 3765.45 cm^−1^. All the nanoplate’s Raman spectrum exhibit L shape behavior with corresponding characteristic peaks except Zn nanoplates display S shape movement which agree with composite’s Raman profile (except CoFC) (Figure 5a,b). In the composites only CoFC and FeFC exhibit downward characteristic peaks at 271.42, and 284.41 cm^−1^, respectively. The peaks at 526.41, 676.1, 802.77, 1085.71, 1340.69, and 1579.22 cm^−1^ indicate presence of CoFC and CuFC generate peaks at 426.06, 764.93, 1062.68, 1135.06, 1307.79, 1450.90, 2724.15, 2857.4, 2885.36, 2929.78, and 3035.06 cm^−1^. The engendered Raman shift at 227.27, 266.23, 354.54, 528.57, 590.91, 646.75, 1080.51, 1276.62, 1311.68, and 1570.13 cm^−1^ imply the FeFC while peaks at 564.24, 797.83, 891.6, 975.49, 1062.68, 1135.1, 1301.21, 1450.9, 2730.74, 2852.47, 2885.37, and 3035.1 cm^−1^ infer NiFC. The ZnFC creates peaks at 952.46, 1062.68, 1135, 1301.21, 1445.97, 2127.1, 2852.46, 2885.36, and 3040 cm^−1^ in which 2127.1 cm^−1^ is the middle point of characteristic arc shape of ZnFC (Figure 5c). It is apparent that the Raman peaks of the nanoplates are shifted and disappeared, which confirms that good bonding occurred in the composite. It is considered that the PVDF is a polar polymer and the nanoplate surface can interact well by dipolar interaction with binder and form good composites [13].

### 3.5. Hydrophobic Property and Electric Conductivity

**Figure 6:**
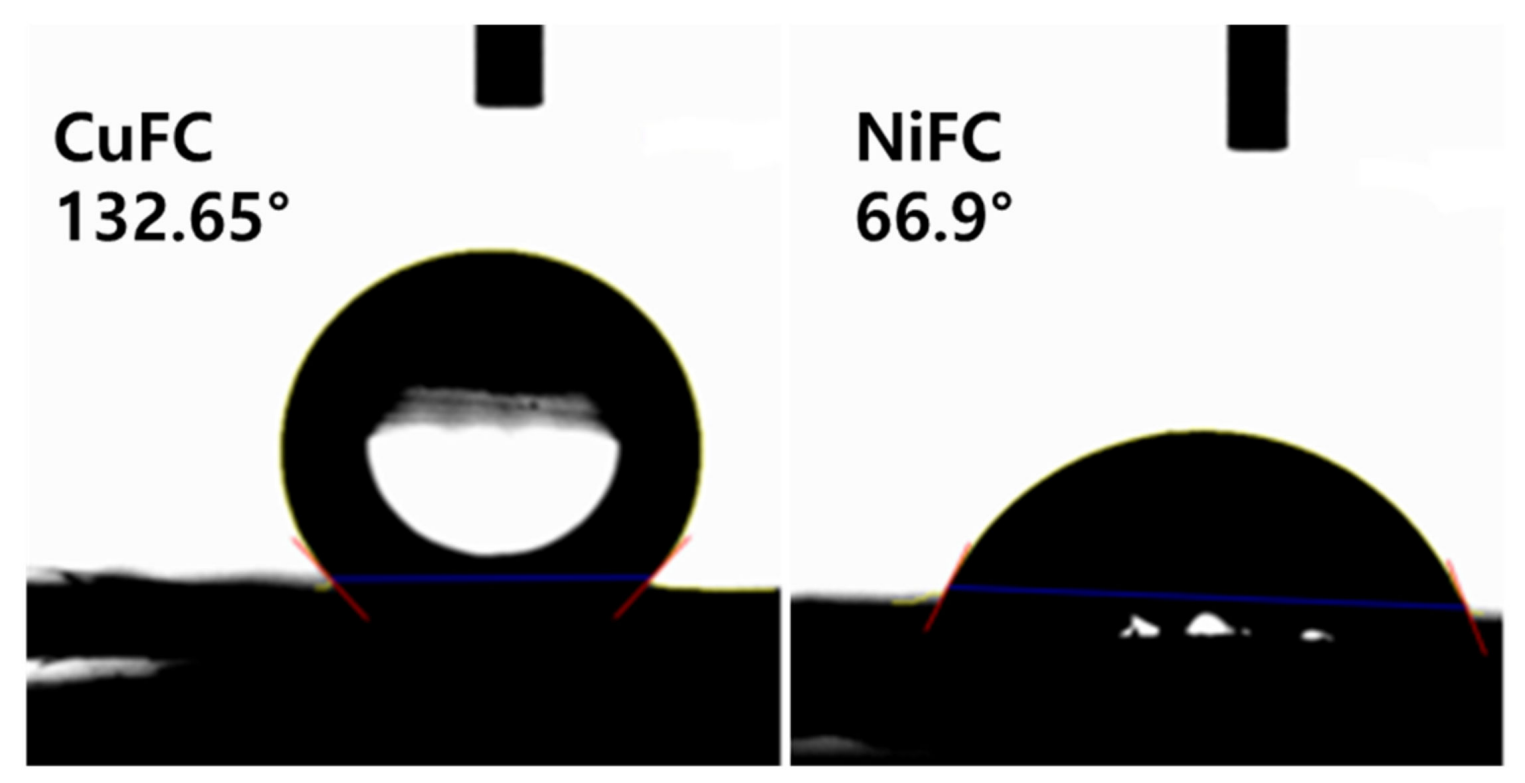
Hydrophobic and hydrophilic property of CuFC and NiFC.

The contact angle measurement enables to predict the wetting ability of the surface (hydrophilic and hydrophobic nature). The contact angle above 90° is considered a hydrophobic surface while below 90° is denoted as hydrophilic surface, and a contact angle above 150° considered a super hydrophobic surface. It is remarkable that the surface physio-chemical properties of the materials determine the wetting and non-wetting ability of the surface. The various materials, such as nanoparticles, ligands, and polymers, are being used to create hydrophobic and hydrophilic surface. In addition, the water hating materials increase contact angle whereas water loving (polar materials) significantly improve hydrophilic nature. Furthermore, increasing the surface roughness, and groves, rise the surface energy, although, surface morphology, and chemical composition also affect the wettability of the surface [13,25,26]. The hydrophobic composite is an attractive candidate in the microelectronic device and material with other properties such as oxidation resistance, antifouling, self-cleaning, are highly preferable as these properties improve lifespan of the electronic devices [27]. In generally, the composite prepared are hydrophobic except NiFC. The composites CoFC, CuFC, FeFC, NiFC, and ZnFC exhibit a contact angle of (118.09, 132.65, 120.6, 66.90, and 116.60)° while show (−34.28, −49.32, −37.06, 28.56, and −32.60) mN/m of wetting energy, respectively. It is obvious that the wetting energy is increased when hydrophilic nature of the surface increase whereas reducing work of adhesion rise the wetting ability of the surface. The CoFC, CuFC, FeFC, NiFC, and ZnFC composites display spreading coefficients of −107.08, −122.12, −109.86, −44.24, and −105.40 mN/m and decreasing spreading coefficient improve the hydrophobic properties of the surface. It is obvious that the composition of the composites considerably improves surface roughness and hydrophobic property (Figure 2a–f and Figure 6, and Table 2). The electrical conductivity (σ) which is due to the mobile charges present in the composite and inversely proportional to the resistivity (ρ). For the composite the resistivity depends on the sheet resistance (*Rs*) and thickness of the composite (t) [6]. The σ range of the composite is 3.461–22.731 S.cm^−1^ while *Rs* ranging from 2.889–11.89 ohm/sq. The cobalt based composite showed excellent EC which is due to the excellent alignment of the Co nanoplate while ZnFC shows lower EC among prepared composites. The higher the sheet resistances, the higher the EC, but the thickness of the composite plays a crucial role in EC. Hence, introduction of the long metal nanoplate greatly improve the EC while ZnFC considerably reduce EC. Furthermore, the alignment of the nanoplate in the composite also affects the EC and sheet resistance (Figure 1, Figure 2 and Table 3).

(1) $$\sigma =\frac{1}{\rho }={\left(Rs.t\right)}^{-1}$$

(2) ρ=Rs.t

(3) $$R=\rho \frac{L}{A}=\rho \frac{L}{wt}=Rs\frac{L}{w}$$

A = wt(4)

(5) $$\mathrm{Rs}=\frac{\rho }{t}$$

### 3.6. EMI Shielding of the Composites

#### 3.6.1. EMI Shielding Theory and Mechanisms

The propagating electromagnetic radiation (EMR) can be weaken in the various frequency range (L, S, C, X, K, and Ku bands) which depends on the type of the shielding materials used. The specific power of EMR (P_I_) colloids on the surface of the shielding materials experience different alterations such as transmittance (P_T_), and absorption (P_A_), and reflection (P_R_). The reflection (R) occurs on the surface while absorption (*A*) happens within the materials which depends on the type of material used and remaining come out as transmittance EMR. The A and absorption coefficient (*Ae*) can be expressed as follows, where reflection (*R*) and transmittance (*T*) are correlated (Equations (6) and (7)).

(6) A=1−R

(7) $$A_{e}=\left[\frac{1-R-T}{1-R}\right]$$

The diminution of EMR is articulated by shielding effectiveness (SE) and its unit is dB. According to the EMI shielding theory, the SE could be defined logarithmic ratio between *P_i_* and power of transmittance (*P_t_*). In addition, it can also be defined by using electric intensity (*E*), magnetic intensity (*H*), wavelength (*λ*), and slot length (*l*) of the EMR. The total shielding effectiveness (*SE_T_*) is stated by using the following equations, where *i* and *t* are denoted as incident and transmittance wave, respectively (Equation (8)).

(8) $$SE_{T}=10log\left(\frac{P_{i}}{P_{t}}\right)=20log\left(\frac{H_{i}}{H_{t}}\right)=20log\left(\frac{E_{i}}{E_{t}}\right)=20log\left(\frac{\lambda }{2l}\right)$$

Further, the sum of reflection (*SE_R_*), absorption (*SE_A_*) and multiple reflection (*SE_MR_*) give rise to *SE_T_* (Equation (9)).

(9) $$SE_{T}=SE_{R}+SE_{A}+SE_{MR}$$

If the *SE_T_* is very small (>15 dB), the *SE_MR_* can be neglected and equation can be written as follows (Equation (10))

(10) $$SE_{T}=SE_{R}+SE_{A}$$

Moreover, the T and R depend on the electric intensity (*E*) and scattering parameters, and can be expressed Equations (11) and (12) in which *t* is transmittance wave, *i* is incident wave and *r* is reflection wave.

(11) $$T={\left|S_{12}\right|}^{2}={\left|\frac{E_{t}}{E_{i}}\right|}^{2}={\left|S_{21}\right|}^{2}$$

(12) $$R={\left|S_{11}\right|}^{2}={\left|\frac{E_{r}}{E_{i}}\right|}^{2}={\left|S_{22}\right|}^{2}$$

Further, the *SE_T_*, *SE_R_*, and *SE_MR_* can be expressed in terms of scattering parameters, wave impedance of air (*Z_o_*), propagation constant (*β*), wave impedance of material (*Z_m_*), thickness of the shielding materials (*t*), relative magnetic permeability (*µ_r_*), and imaginary unit (*j*) (Equations (13)–(15)).

(13) $$SE_{T}=10\log\left(T\right)=10log\left(\frac{1}{1-{\left|S_{12}\right|}^{2}}\right)=SE_{R}+SE_{A}=10log\left(\frac{1}{{\left|S_{21}\right|}^{2}}\right)$$

(14) $$SE_{M}=20\log\left(\frac{1}{4}\;\sqrt{\frac{\sigma }{\omega \mu_{r}\varepsilon_{o}}}\right)=20log\left|1-e^{-2t/\delta }\right|=20\log\left|1-{\left(\frac{Z_{o}-Z_{m}}{Z_{o}+Z_{m}}\right)}^{2}e^{-2t/\delta }e^{-2j\beta t}\right|$$

(15) $$SE_{R}=10\log\left(1-R\right)=20\;log\left|\frac{{\left(Z_{o}+Z_{m}\right)}^{2}}{4Z_{o}Z_{m}}\right|=\left(\frac{1}{1-{\left|S_{11}\right|}^{2}}\right)≅20log\left|\frac{Z_{o}}{4Z_{m}}\right|$$

In addition, the *t*, skin depth (*δ*), relative conductivity (*б_r_*), *µ_r_*, refractive index (n), and imaginary part of wave vector (ik) also influence *SE_A_*, *SE_R_*, and *SE_MR,_* and expressed in following Equations (16)–(18).

(16) $$SE_{A}=\frac{8.7t}{\delta }=10log\left(1-A_{e}\right)=131.4d\sqrt{f\mu_{r}\sigma_{r}}=K\left(\frac{t}{\delta }\right)=10log\left[\frac{T}{\left(1-R\right)}\right]=20lm\left(k\right)d\;log\;e=20loge^{t/\delta \;}$$

(17) $$SE_{M}=168+10log\left(\frac{\sigma_{r}}{\mu f}\right)=20\log\left|1-10^{\frac{SE_{A}}{10}}\right|=20log\left|\frac{1-\left(1-n^{2\;}\right)}{{\left(1+n\right)}^{2}}\exp\left(2ikd\right)\right|$$

(18) $$SE_{R}=39.5+10log\left(\frac{\sigma }{2\pi f\mu }\right)=108+\log\left(\frac{\sigma }{f\mu }\right)=20log\left|\frac{1+n^{2}}{4n}\right|$$

Skin depth is inversely proportional to square root of πfбμ of the composite where *μ* is magnetic permeability, *f* is frequency of *EM_R_*, and *б* is the electric conductivity (Equation (14)).

(19) $$\delta =\frac{1}{\sqrt{\pi f\sigma \mu }}$$

The propagating EMR undergoes the changes along the direction of wave (near field (r < λ/2π) to far field (r>λ/2π)). Therefore, most of the EMR are far field and planar waves. The intrinsic impedance of the wave Z can be expressed that the amplitude ratio between electric fielding (*E*) and magnetic field (*H*) waves (*E*┴*H*). Moreover, *б*, *μ*, angular frequency (*ω = 2πf*), *j*, and electric permeability (*ε*) influence the *Z*. *Z_o_* is *Z* in air, and has the value of 377 Ω (*j* = *ω* = 1 and б = 0) (Equations (20)–(22)).

(20) $$Z=\frac{\left|E\right|}{\left|H\right|}$$

(21) $$Z_{o}=\sqrt{\frac{\mu_{o}}{\varepsilon_{o}}}$$

(22) $$Z=\sqrt{\frac{j\omega \mu }{\sigma -j\omega \varepsilon }}$$

The physiochemical properties of individual composition of the composite determine the EMI shielding. The Maxwell Garnett formula is used to calculate effective relative permittivity *ε_eff_* which can be determined by using relative permittivity of the matrix (*ε_e_*), *f* is volume fraction of the filler, and relative permittivity of the fillers (*ε_i_*). The *ε_i_* is calculated by using an imaginary part of the complex relative permittivity (*ε*′and *ε*″), imaginary unit (j), *б*, *ω*, and *ε_o_* (Equations (23) and (24)).

(23) $$\varepsilon_{i}=\varepsilon^{\prime }-j\varepsilon^{\prime \prime }=\varepsilon^{\prime }-j\frac{\sigma }{\omega \varepsilon_{o}}$$

(24) $$\varepsilon_{eff}=\varepsilon_{e}+3f\varepsilon_{e}\frac{\varepsilon_{i}-\varepsilon_{e}}{\varepsilon_{i}+2\varepsilon_{e}-f\left(\varepsilon_{i}-\varepsilon_{e}\right)}$$

On the other hand, transmittance of the EMR depend on the transmission coefficient (0-t boundary (*T*_1_ and *T*_2_)), reflection coefficient (0-t boundary (*R*_1_ and *R*_2_)) in which 0 is considered as 1 and t = 2, and complex propagation constant (*γ_m_*). The *μ*, *ε*, *j*, and ω affect the value of *γ_m_* of the composite (Equations (25) and (26)).

(25) $$\gamma_{m}=j\omega \sqrt{\varepsilon_{o}\mu_{o}(\varepsilon_{eff}^{\prime }-j\varepsilon_{eff}^{\prime \prime }}$$

(26) $$T=\;\frac{T_{1}T_{2}e^{-\gamma_{m}D}}{1+R_{1}R_{2}e^{-2\gamma_{m}D}}$$

**Scheme 1:**
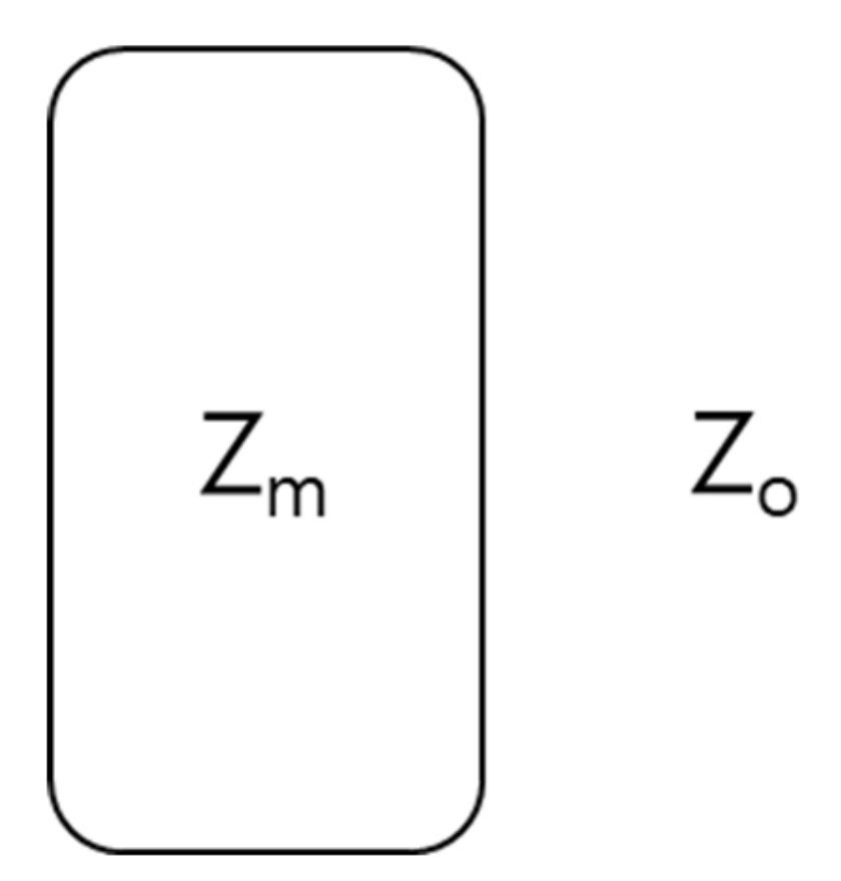
Representation of the *Z_o_* and *Z_m_* in a composite.

*Z_o_* and *Z_m_* determine the magnitude of *T*_1_ and *R*. Moreover, *Z_o_*, *μ_r_*, and *ε_eff_* have an impact on the value of *Z_m_* (Scheme 1 and Equations (27)–(31)).

(27) $$T_{1}=\frac{2Z_{m}}{Z_{m}+Z_{o}}$$

(28) $$R_{1}=\frac{Z_{m}-Z_{o}}{Z_{m}+Z_{o}}$$

(29) $$T_{2}=\frac{2Z_{o}}{Z_{m}+Z_{o}}$$

(30) $$Z_{m}=Z_{o}\sqrt{\frac{\mu_{r}}{\varepsilon_{eff}}}$$

(31) $$R_{2}=\frac{Z_{o}-Z_{m}}{Z_{m}+Z_{o}}$$

Hence, The SE can be calculated in terms of T and shielding efficiency (%) of the materials can be calculated according to Equation (32) [6].

(32) $$\mathrm{SE}=-20\log\left(\left|T\right|\right)$$

(33) $$\mathrm{Shielding}\;\mathrm{efficiency}\;\left(\%\right)=100-\;\left(\frac{1}{10^{SE/10}}\right)\times 100$$

#### 3.6.2. EMI shielding of Composites

The study shows that the composites have shielding efficiency ranging from 99.9913–99.99964% in which CoFC exhibits the excellent EMI SE among composites prepared. Han et al. reported that the spray coated Ti_3_C_2_T_x_-MXene layer with 40 nm exhibits 21 dB of EMI SE and most of the MXene with thickness below 10 micron and above skin depth shows EMI SE above 20 dB. Further he added that the Ti_3_C_2_T_x_ MXene displayed good EMI shielding efficiency among other MXenes [28]. Chen et al. explained that the introduction of the silver nanowire considerably improves EMI SE and added that the neat silver nanowire gives rise 21 dB while MXene-welded silver nanowire film exhibits 34 dB with excellent mechanical properties. However, the microstructure of the composites influences the EMI SE, which depends on the constitutional elements of the composites [29]. The core-shell Fe_3_O_4_@SnO_2_-epoxy composite exhibits good microwave absorption of −36.5 dB with the thickness of 2 mm. In addition, the high porosity, core-shell structure and magnetic behavior of the composite, are the reason for the good microwave absorption. Moreover, these parameters improve dipolar nature that is the main reason for the microwave absorption [30]. The study by Zhao et al. showed that Ni/SnO_2_ show similar microwave absorption like Fe_3_O_4_@SnO_2_ with 1.7 mm of thickness. In both cases, composite possess micro pores, however, the thickness is different. It is apparent that the magnetic Fe_3_O_4_ and epoxy resin enhance the EMI SE significantly [30,31]. According to the Fei et al. the C/Co/cellulose nanofiber composite displays EMI SE of 35.1 dB with the lower density of 0.00174 g cm^−3^ which is due to the aerogel structure. The aerogel structure is created by heat treatment while Ni/SnO_2_ consist micro pores formed due to the acid treatment which also induces microwave absorption [31,32]. Moreover, various metal oxide, and sulfide with various polymers have been used as shielding materials and shielding efficiency has been tuned by changing the composition in the shielding materials. The band gap of the semiconductors generally lies between the 1–1.5 eV and it can be increased 2–4 eV by changing the particle size. The various hybrid composites can be prepared by mixing carbon, polymer materials, and nanoparticles (MoS_2_, CoO_3_, Fe_3_O_4_, ZnO, and CuS), and those reported are used to improve EMI SE of the composite [33]. The microwave absorbing graphene epoxy composite prepared by solution mixing process and corresponding composite consists 95% of the carbon and remaining are oxygen which exhibits EMI SE of −29 dB with 2 mm thickness and 2.5 wt.% of the graphene content [34]. Rajavel et al. report showed that the intercalation of silver into the Nb_2_CT_x_ MXene greatly improve the EMI SE of paraffin composite with thickness of 1 mm. Further he stated that pure Nb_2_CT_x_ (60%) in paraffin showed EMI SE of 12.96 dB while Ag.Nb_2_CT_x_ with the same ration exhibited 72.04 dB which is due to higher EC, surface oxides (Nb_2_O_5_), interfacial heterogenous surface, and induced polarization loss [35]. It is obvious that the introduction of metal nanomaterials improves the EMI SE, because they alter the shielding parameters. Thus, this study focuses on the preparation of the cost effective nanoplate composite for the EMI SE application. 

**Figure 7:**
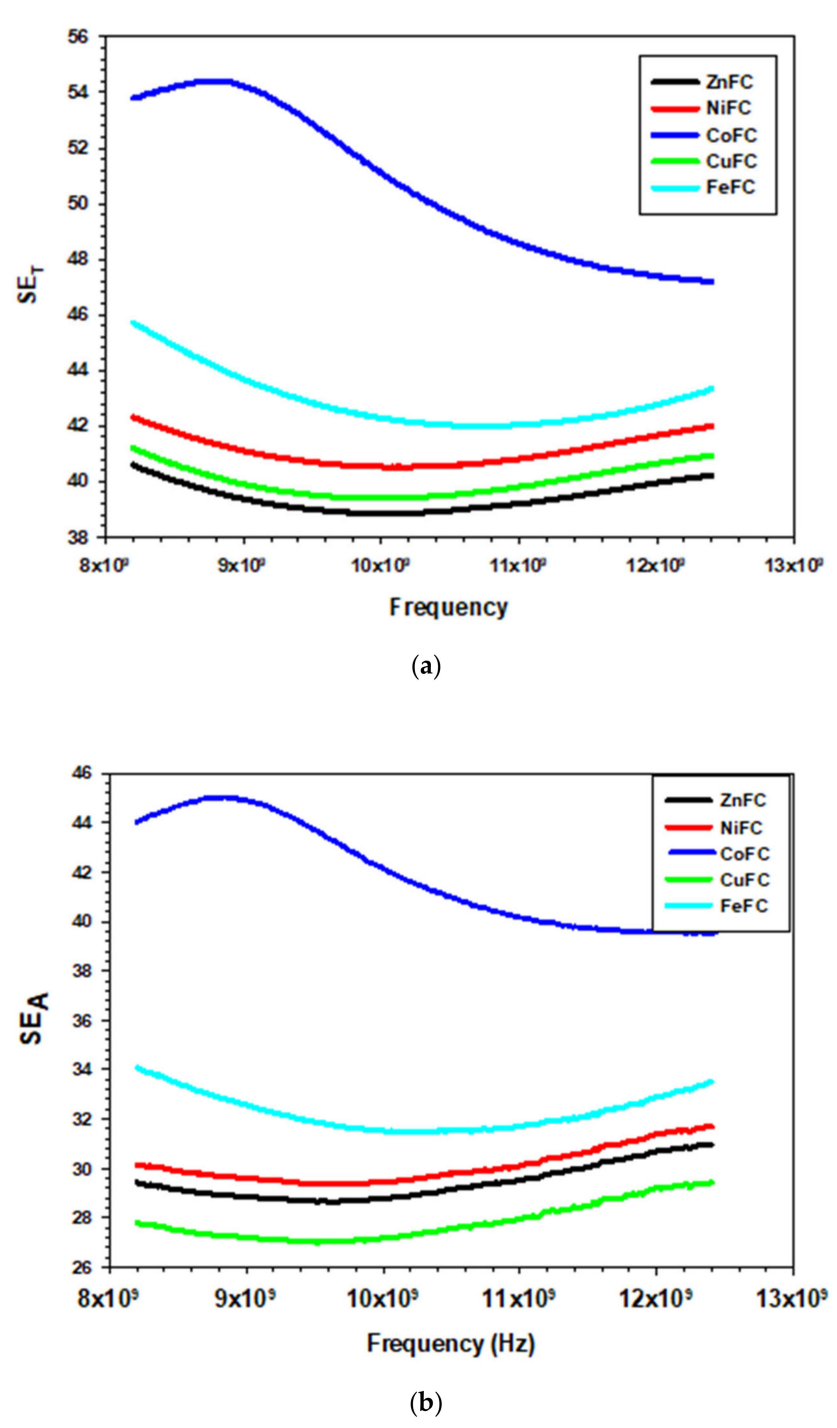

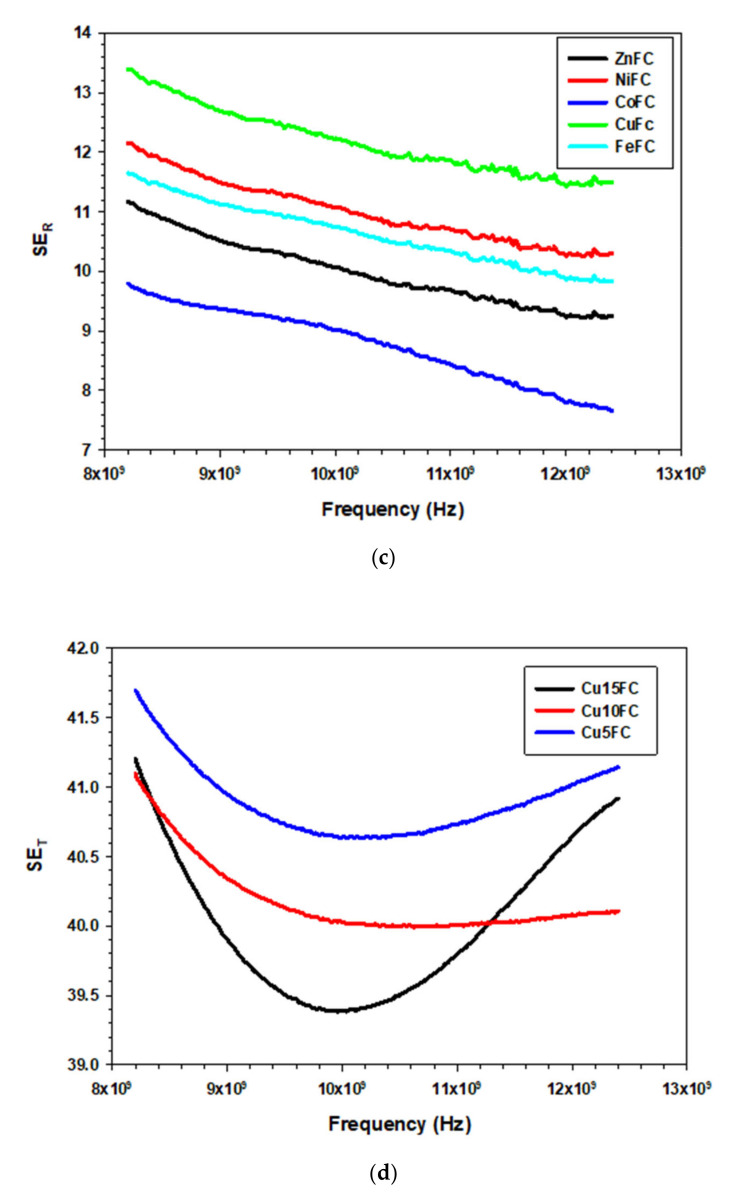
EMI shielding of the composite (**a**) SE_T_, (**b**) SE_A_, (**c**) SE_R_, and (**d**) differently coated Cu nanoplate.

The composites ZnFC, NiFC, CoFC, FeFC, and CuFC show maximum EMI SE of 40.59, 42.31, 54.43, 45.72, and 41.21 dB, respectively and corresponding minimum EMI SE are 38.85, 40.51, 47.19, 41.98, and 39.38 dB, respectively. In addition, the composites showed average EMI SE of 39.41, 41.10, 50.64, 42.91, and 40.00 dB, respectively, and corresponding composites are ZnFC, NiFC, CoFC, FeFC, and CuFC, respectively. Among those, the CoFC exhibits higher EMI SE, which is due to the higher EC of the composite and all the composites thickness are within the 400 µm (Figure 6 and Table 3, Table 4 and Table 5). The CoFC shows the higher SE_A_ (45 dB) which about 82.69% of the total EMI SE and remaining is SE_R_ (9.79 dB). This imply that the porous structure of the CoFC played a major role in EMI SE and EC has less significant contribution for the EMI SE. ZnFC, NiFC, FeFC, and CuFC exhibited SE_A_ of 30.97, 31.67, 34.07, and 29.41 dB, respectively, while the corresponding composites showed SE_R_ of 11.16, 12.15, 11.65, and 13.39 dB, respectively (Figure 7a–d). The SE/d ranging from 101.99–147.11 dB mm^−1^ in which CoFC showed higher SE/d while ZnFC exhibited lowest SE/d (Table 5). The all the composite coated 15 times, However, Cu based composites were prepared for different coating cycle. The composites denoted as Cu5FC, Cu10FC and Cu15FC (CuFC) indicating that 5, 10, and 15 times coated composites, respectively. It is noticeable that the all the composites prepared by using Cu nanoplate showed almost same EMI SE (99.99%). Hence, the nanoplates are more effective EMI shielding materials even at lowest amount. The maximum EMI SE of Cu5FC, Cu10FC, and Cu15FC are 41.21, 41.09, and 41.69 dB, respectively, in which Cu5FC shows slightly higher EMI SE than others which confirmed that little amount of nanoplate is enough for the better EMI shielding application (Figure 6d). General EMI shielding mechanism of the composites can be descripted as follow that the little amount of the incoming radiation reflected on the surface and entering radiation into the composite matrix undergo multiple reflection which finally leads to absorption. The polypropylene/CNT composite blocks about 99.9% of the incident wave with 2 cm of thickness and 14.4 S m^−1^ of EC, and most prominently the incident wave undergo absorption. It is noteworthy that the distribution of the conductive CNT network in the composite play major role in the shielding behavior and It is also mentioned that the absorbed energy is converted into heat [36]. The graphene is a two-dimensional material has been utilized for the EMI shielding application for which graphene is mixed with different polymers to achieve excellent EMI SE. The graphene/Polydimethylsiloxane (PDMS) composite with 0.42 wt.% of filler loading has been reported by Gao et al. exhibits EMI SE of 63 dB and composites possess nacre-mimetic aligned lamellar structure with three-dimensional network and lowest threshold loading. Further, the graphene/PDMS hold aerogel structure with the lower EC (0.32–0.007 S m^−1^) in which aerogel structure plays major role in the EMI SE [37]. The MXene and metal composite films with lower thickness and various materials showed excellent EMI SE compare to other materials used such as rGO, metal nanoparticle and oxides, and CNT with other binder showed above 99% shielding efficiency. Further, lower temperature for the particular materials greatly improve EMI SE (Table 5). Thus, the cost-effective nanoplate is one of the good choices for the EMI shielding application in various application domains.

## 4. Conclusions

Composites were successfully prepared by a cost-effective spray coating technique with light weight and lower thickness range of 0.282–0.398 mm. The non-woven carbon fabric was exploited as the template for the spray coating process. The fabricated composites are amorphous in nature and have a non-wetting surface. The contact angle ranged from 132.65 to 66.90° and the wetting energy of the composites ranged from 25.56 to 49.32 mN/m. The CuFC have excellent hydrophobic nature with contact angle of 132.65°, and wetting energy of −49.32 mN m^−1^. The spreading coefficient range of the composites is from −122.12 to –44.24 mN m^−1^ and the work of adhesion range of the composites is −101.36–23.48 mN m^−1^. Among the composites, the NiFC has the lowest spreading coefficient value while CuFC has the lowest work of adhesion. The sheet resistance, resistivity, and electrical conductivity range of the composites are 2.889–11.890 ohm sq^−1^, 0.1115–0.4399 Ω cm^−1^, and 3.461–8.967 S cm^−1^, respectively. The EMI SE range of the composites is 40.59–54.43 dB and corresponding shielding efficiency is 99.9913–99.99964%. The CoFC holds the highest EMI SE, EMI shielding efficiency, and SE/d, and ZnFC takes a lower value. Thus, the composites can be utilized in various electronic domains.

## Figures and Tables

**Table 1:** Atomic percentage of the composite from XPS.

Composite	C1s	O1s	F1s	N1s	S2p	Metal 2p
ZnFC	79.90	11.32	2.11	2.75	3.08	0.84
FeFC	78.93	12.02	1.93	2.60	3.03	1.48
NiFC	68.74	12.36	12.18	2.00	2.26	2.00
CoFC	56.84	17.34	15.93	1.66	1.71	5.36
CuFC	82.23	10.37	1.16	2.81	3.05	0.39

**Table 2:** Water repellent properties of the composite.

Composite	Contact Angle	Wetting Energy [mN/m]	Spreading Coefficient [mN/m]	Work of Adhesion [mN/m]
CoFC	118.09	−34.28	−107.08	38.52
CuFC	132.65	−49.32	−122.12	23.48
FeFC	120.60	−37.06	−109.86	35.74
NiFC	66.90	28.56	−44.24	−101.36
ZnFC	116.60	−32.60	−105.40	40.20

**Table 3:** Electric conductivity of the composites.

Parameters	CoFC	CuFC	FeFC	NiFC	ZnFC
Sheet resistance (ohm/sq)	11.890	4.125	2.889	6.175	7.260
Thickness (cm)	0.0370	0.0282	0.0386	0.0330	0.0398
resistivity (ρ=Rs.t) (Ω.cm)	0.4399	0.1163	0.1115	0.2038	0.2889
EC (σ = 1/(Rs.t)) (S.cm^−1^)	2.2731	8.597	8.967	4.907	3.461

**Table 4:** Maximum, average, minimum EMI SE, and maximum shielding efficiency (SE) (%) comparison for the composites.

Composites	ZnFC	NiFC	CoFC	FeFC	Cu15FC	Cu10FC	Cu5FC
MAX (dB)	40.59	42.31	54.43	45.713	41.21	41.09	41.69
Ave (dB)	39.41	41.09	50.63	42.91	40.00	40.18	40.89
Min (dB)	38.84	40.51	47.19	41.98	39.39	39.99	40.63

**Table 5:** Comparison of EMI shielding of different composites.

No.	Composite	Thickness (mm)	EC (S.cm^−1^)	SE (dB)	SE (%)	SE/d (dB.mm^−1^)	Ref.
1	Metal-Wrapped Cellular Membranes	0.005	*	76.8	99.9999979	15360	[38]
2	PEDOT:PSS/Ti_3_C_2_T_x_	0.006	*	41	99.9921	6833.33	[39]
3	AgNW-modified textile	1.4	*	55	99.99968	39.29	[40]
4	Fe nanoparticles and CNTs co-decorated porous carbon/graphene foam	1	*	48	99.9984	48	[41]
5	rGO/sugarcane	3	6	53	99.99949	17.67	[42]
6	liquid metal composites	3	*	75 (77 K) 21.51 (300 K)	99.9999968	25	[43]
7	Titanium Carbide (MXene) Nanocoatings	0.001	2900	52	99.99937	52000	[44]
8	carbon-fabric/Ag/waterborne polyurethane	0.183	11986.8	102.8	99.99999999	561.75	[9]
9	PET Fiber@Microporous Organic Polymer with Amino Groups@Cu Films	0.00064	*	73.8	99.9999958	114062.5	[45]
10	polyurethane/MXene nacre-like nanocomposite films	0.0074	5983.5	61.4	99.999928	8297.30	[46]
11	Aramid Nanofiber–Ti_3_C_2_T_x_ MXene/Silver Nanowire Nanocomposite	0.091	3725.6	79.8	99.9999989	876.92	[47]
12	Conductive MXene-Based Films	0.007	2638.83	50.17	99.99904	7244.29	[48]
13	Reduced graphene oxide/zinc oxide coated conductive cotton textile	0.00439	15.79	54.9	99.99968	12505.69	[49]
14	ANF/MXene composite	0.0032	879	40.6	99.9913	12687.5	[50]
15	MXene- Aramid Nanofiber composite	0.02137	36.618	34.6	99.9653	1616.10	[51]
16	PDMS-coated nickel ferrite/MWCNT/cotton fabrics	0.32	0.18	37	99.9800	115.63	[52]
17	Fe_3_O_4_/C/PPy core/shell composites	0.8	*	28	99.8415	35	[53]
18	Cellulose Nanofibers/Ti_3_C_2_T_x_ MXene Aerogels/Epoxy Nanocomposites	2	16.72	74	99.9999961	37	[54]
19	MXene-poly(3,4ethylenedioxythiophene): poly(styrene sulfonate) (PEDOT:PSS)	0.006	*	41	99.9921	7666.67	[39]
20	Zn^2+^/Ti_3_C_2_T_x_ MXene-Based Foam	0.085	56.718	51	99.99921	600	[55]
21	ZnFC	0.398	3.461	40.59	99.9913	101.99	This work
22	NiFC	0.330	4.907	42.31	99.9941	128.21	
23	FeFC	0.386	8.967	45.72	99.9973	110.67	
24	CuFC	0.282	8.597	41.21	99.9924	146.14	
25	CoFC	0.37	22.731	54.43	99.99964	147.11	

*: There is no information or impossible to calculate.
